# PleThora: Pleural effusion and thoracic cavity segmentations in diseased lungs for benchmarking chest CT processing pipelines

**DOI:** 10.1002/mp.14424

**Published:** 2020-08-28

**Authors:** Kendall J. Kiser, Sara Ahmed, Sonja Stieb, Abdallah S. R. Mohamed, Hesham Elhalawani, Peter Y. S. Park, Nathan S. Doyle, Brandon J. Wang, Arko Barman, Zhao Li, W. Jim Zheng, Clifton D. Fuller, Luca Giancardo

**Affiliations:** ^1^ John P. and Kathrine G. McGovern Medical School Houston TX USA; ^2^ Center for Precision Health UTHealth School of Biomedical Informatics Houston TX USA; ^3^ Department of Radiation Oncology University of Texas MD Anderson Cancer Center Houston TX USA; ^4^ MD Anderson Cancer Center‐UTHealth Graduate School of Biomedical Sciences Houston TX USA; ^5^ Department of Radiation Oncology Cleveland Clinic Taussig Cancer Center Cleveland OH USA; ^6^ Department of Diagnostic and Interventional Imaging John P. and Kathrine G. McGovern Medical School Houston TX USA

**Keywords:** computer‐aided decision support systems, image segmentation techniques, image processing, informatics in imaging, quantitative imaging

## Abstract

This manuscript describes a dataset of thoracic cavity segmentations and discrete pleural effusion segmentations we have annotated on 402 computed tomography (CT) scans acquired from patients with non‐small cell lung cancer. The segmentation of these anatomic regions precedes fundamental tasks in image analysis pipelines such as lung structure segmentation, lesion detection, and radiomics feature extraction. Bilateral thoracic cavity volumes and pleural effusion volumes were manually segmented on CT scans acquired from The Cancer Imaging Archive “NSCLC Radiomics” data collection. Four hundred and two thoracic segmentations were first generated automatically by a U‐Net based algorithm trained on chest CTs without cancer, manually corrected by a medical student to include the complete thoracic cavity (normal, pathologic, and atelectatic lung parenchyma, lung hilum, pleural effusion, fibrosis, nodules, tumor, and other anatomic anomalies), and revised by a radiation oncologist or a radiologist. Seventy‐eight pleural effusions were manually segmented by a medical student and revised by a radiologist or radiation oncologist. Interobserver agreement between the radiation oncologist and radiologist corrections was acceptable. All expert‐vetted segmentations are publicly available in NIfTI format through The Cancer Imaging Archive at https://doi.org/10.7937/tcia.2020.6c7y‐gq39. Tabular data detailing clinical and technical metadata linked to segmentation cases are also available. Thoracic cavity segmentations will be valuable for developing image analysis pipelines on pathologic lungs — where current automated algorithms struggle most. In conjunction with gross tumor volume segmentations already available from “NSCLC Radiomics,” pleural effusion segmentations may be valuable for investigating radiomics profile differences between effusion and primary tumor or training algorithms to discriminate between them.

## 1 INTRODUCTION

Automated or semi‐automated algorithms aimed at analyzing chest computed tomography (CT) scans typically require the creation of a three‐dimensional (3D) map of the volume‐of‐interest (VOI) as the initial step.^1^, ^2^ For example, a common first step for lung tumor detection on CT scans is lung segmentation.^3^ Effective strategies for identifying healthy lungs have existed at least since Hu et al. introduced a method for segmenting healthy lung parenchyma based on gray‐level thresholding.^4^ Nevertheless, identifying the initial VOI in pathologic lungs remains an obstacle.^5^, ^6^ Many pathologic states — such as pleural effusion, severe fibrosis, or tumor — can alter the space lung would normally occupy, and in this circumstance the VOI is not only the lung but the thoracic cavity. To build image processing pipelines intended to analyze chest CTs with substantively altered thoracic anatomy, identifying this VOI is critical.

Preprocessing strategies to identify thoracic VOIs in the presence of pathology have been described. In 2014, Mansoor et al. presented a seminal approach that identified CTs with large volumetric differences between autosegmented lung and the thoracic cage and refined these lung segmentations using texture‐based features.^7^ Subsequent studies have approached VOI identification in myriad ways, such as threshold‐based methodologies,^8^ deep learning architectures,^9^, ^10^, ^11^, ^12^, ^13^ anatomic or shape‐prior models,^5^, ^14^, ^15^, ^16^ and region‐growing methods.^17^, ^18^

As methodologies to identify thoracic VOIs in pathologic lungs march forward, data to train and vet them must keep pace. Hofmanninger et al. cited a paucity of diverse data — not inferior methodologies — as the principal obstacle in pathologic lung segmentation.^19^ Echoing this point, Shaukat et al. observed that automated lung nodule detection system optimization is usually limited to just one dataset.^20^ Many datasets consist exclusively of diseases typified by mild to moderate anatomic change — for example, chronic obstructive pulmonary disorder or interstitial lung disease.^10^, ^11^ However, disease processes commonly beset by lung consolidations, effusions, masses, and other opacities that dramatically alter the thoracic VOI are radiographically distinct.

We present PleThora, a dataset of ***ple***ural effusion and left and right ***thora***cic cavity segmentations delineated on 402 CT scans from The Cancer Imaging Archive^21^ (TCIA) NSCLC‐Radiomics collection.^22^, ^23^ Many of these cases have dramatic anatomic changes secondary to cancer. Thoracic segmentations include lung parenchyma, primary tumor, atelectasis, adhesions, effusion, and other anatomic variations when present. On scans where effusion is present, separate segmentations labeling pleural effusion alone are also provided. These may serve particular use for correlating effusion radiomics features with clinical data (available in the NSCLC‐Radiomics spreadsheet, “Radiomics Lung1.clinical‐version3‐Oct 2019.csv”) or studying how these features differ between effusion and primary tumor. Thoracic segmentations were generated automatically by a U‐Net based deep learning algorithm trained on lungs without cancer, manually corrected by a medical student, and revised by a radiation oncologist or a radiologist. Pleural effusion segmentations were manually delineated by a medical student and revised by a radiologist. Expert gross tumor volume (GTV) segmentations already provided by the NSCLC‐Radiomics collection informed our segmentations and made possible delineation of pleural effusion volumes that excluded GTV.

## 2 ACQUISITION AND VALIDATION METHODS

### 2.A Segmentation acquisition

Four hundred twenty‐two Digital Imaging and Communications in Medicine (DICOM) chest CT datasets and 318 corresponding “RTSTRUCT” DICOM segmentations (featuring GTVs) were downloaded from the TCIA NSCLC‐Radiomics collection in January 2019. Four hundred and two CT scans were successfully converted from DICOM to Neuroimaging Informatics Technology Initiative (NIfTI) format using a free executable called “dcm2niix”.^24^, ^25^ These 402 scans comprise the dataset upon which our thoracic cavity and pleural effusion segmentations were delineated.

#### 2.A.1 Thoracic cavity segmentation acquisition

After converting each CT dataset to NIfTI format, lungs were automatically segmented by a publicly available 3D U‐Net lung segmentation algorithm^26^ that had been trained on a private dataset of approximately 200 chest CTs acquired from patients without cancer. Lung segmented reasonably well in subjects with minimal anatomic variation due to disease, but poorly in subjects with significant disease. A fourth‐year medical student manually expanded each segmentation to include the thoracic cavity volume normally occupied by healthy lung. Among other anatomic anomalies, the expansion included normal, pathologic, and atelectatic lung parenchyma, pleural effusion, fibrosis, nodules, tumor, and compensatory anatomic variants such as enlarged collateral vessels. It also included major hilar vessels and bronchi that are not always segmented as part of the lung but are a frequent site of pathologic change. Importantly, effort was made to include all primary tumors in the segmentation even if this invaded the mediastinum or extended out of the thoracic cage. “RTSTRUCT” GTV segmentations from the NSCLC‐Radiomics collection assisted in this determination. Nodal disease in the mediastinum was not targeted. Each hemithorax, right and left, was segmented as a separate structure but saved under the same segmentation file.

**Fig. 1:**
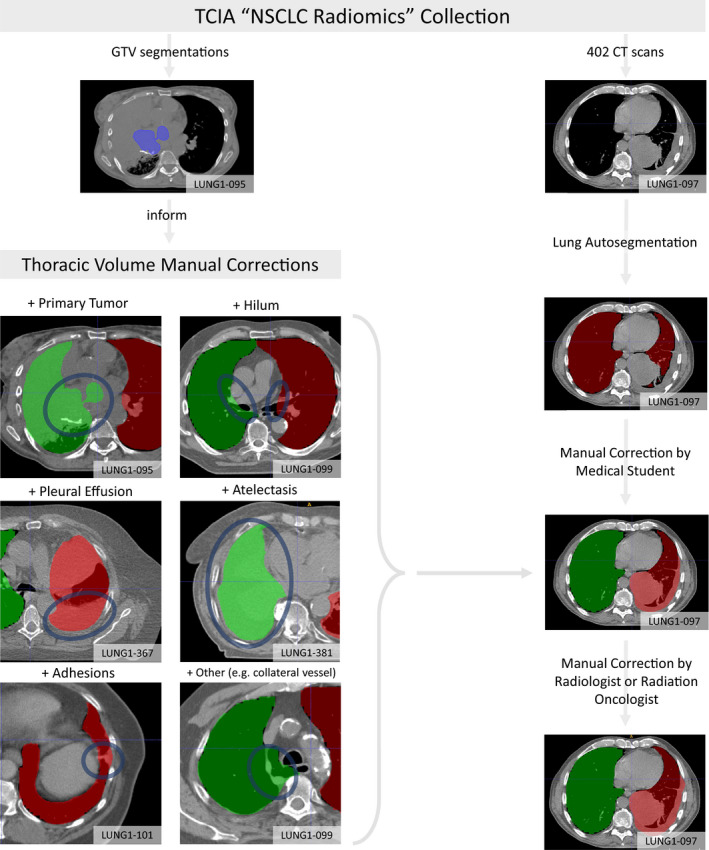
Thoracic cavity volumes were segmented automatically then iteratively corrected by a medical student and at least one radiologist or radiation oncologist to include the entire hemithoraces. [Color figure can be viewed at wileyonlinelibrary.com]

The medical student’s segmentations passed to at least one physician reviewer — either a radiologist or a radiation oncologist — to be vetted. As necessary, reviewers expanded or contracted the segmentations to include the target thoracic volumes. The most recent “RTSTRUCT” GTV segmentations (which were updated by the NSCLC‐Radiomics dataset authors in October 2019) were available to reviewers for reference as necessary. The thoracic cavity segmentations we make public are vetted segmentations. The thoracic cavity segmentation workflow is illustrated in Fig. 1 with examples of various lesions that were included in the delineations.

#### 2.A.2 Pleural effusion segmentation acquisition

**Fig. 2:**
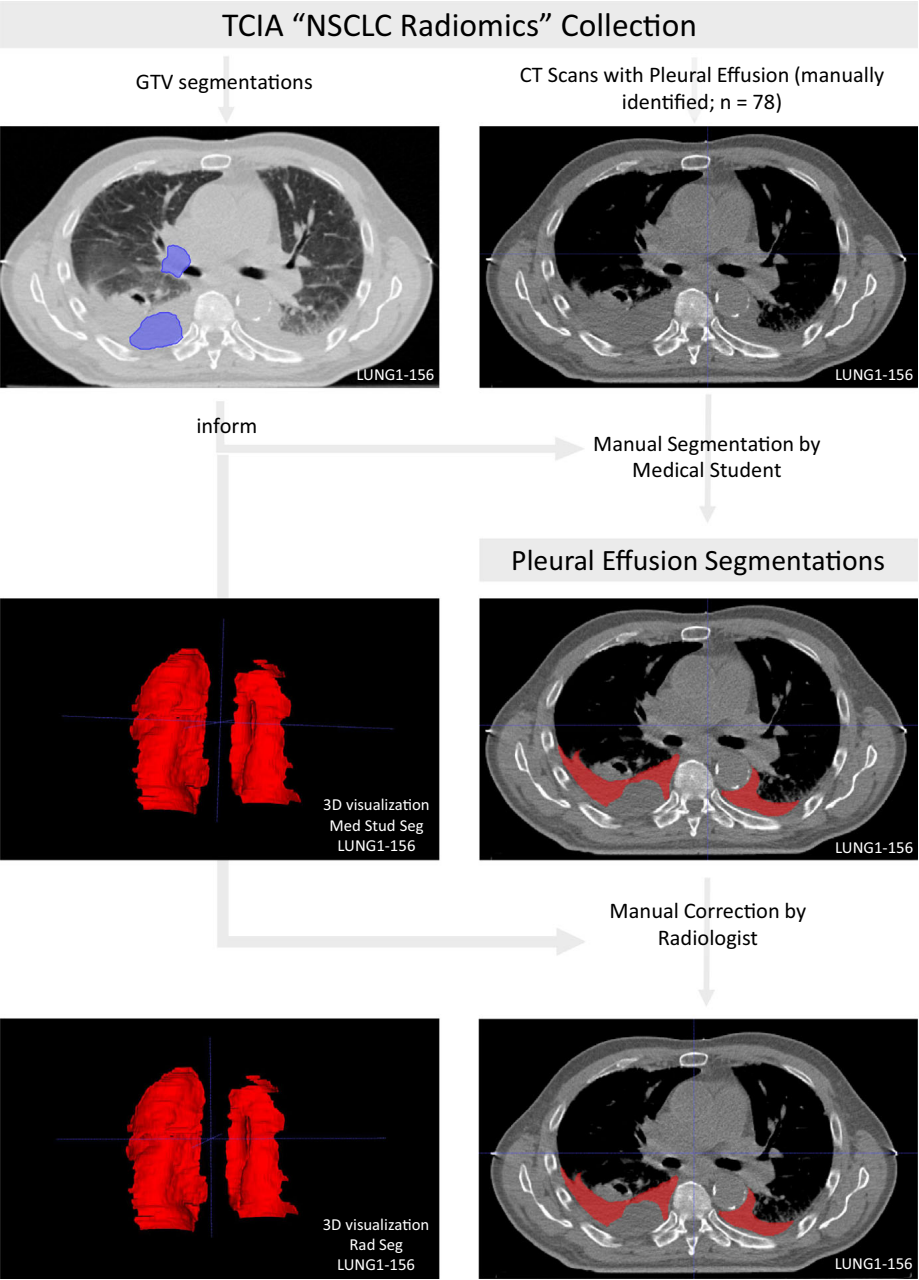
Pleural effusion segmentations, excluding gross tumor volume, were delineated by a medical student and subsequently corrected by at least two radiologists. [Color figure can be viewed at wileyonlinelibrary.com]

A fourth‐year medical student identified a subset of 78 CT scans with trace to massive pleural effusions. The medical student segmented effusions de novo rather than from an autosegmented prior (unlike the thoracic cavity segmentations). Spaces where primary tumor overlapped with effusion were excluded from the segmentation, and NSCLC‐Radiomics GTV segmentations determined the effusion segmentation extent (exemplified in Fig. 2). In most subjects, an effusion in a single hemithorax was segmented. However, effusions were bilateral in 19 subjects, and in this circumstance both sides were segmented and saved under a single structure label.

The medical student's pleural effusion segmentations were vetted by at least two physicians and corrected as necessary. Like the thoracic cavity segmentations, the pleural effusion segmentations we make public are vetted by physicians. The pleural effusion segmentation workflow is illustrated in Fig. 2.

### 2.B Computational tools

Manual segmentations were delineated using ITK‐SNAP v.3.6.^27^ ITK‐SNAP provides tools for manual or semi‐automated segmentation delineation including segmentation interpolation, 3D segmentation visualization and manipulation, and image contrast adjustment permitting mediastinal and lung window visualization. However, ITK‐SNAP does not support structure sets in DICOM‐RT format and could not open “RTSTRUCT” files to view GTV segmentations. Therefore, we used a free DICOM‐RT viewer called Dicompyler^28^ to reference GTV structures while segmenting.

Several additional computational tools transformed CT scans from DICOM to NIfTI format, autosegmented initial thoracic volumes, analyzed segmentations, and organized and visualized metadata. As previously noted, an executable named “dcm2niix”^24^ converted CTs from DICOM to NIfTI format and a 3D U‐Net^26^ automatically generated bilateral lung segmentations. All data were prepared using custom Python scripts leveraging common scientific libraries: NumPy v.1.16.2,^29^ Nilearn v.0.5.0,^30^ Pandas v.0.23.4,^31^ Surface_distance v.0.1,^32^ Scikit‐learn v.0.20.3,^33^ Matplotlib v.3.0.1,^34^ and Seaborn v.0.9.0.^35^

### 2.C Segmentation quality assessment

To assess the quality of segmentations with respect to interobserver variability, volumetric (i.e., traditional) Dice similarity coefficients^36^ (DSC), Cohen’s kappa coefficients,^37^ surface Dice similarity coefficients^32^ (sDSC), 95th percentile Hausdorff distances^38^ (95HD), and symmetric average surface distances (ASD) were computed between expert reviewer segmentations. These metrics are discussed in the following subsections, and lung segmentation interobserver variability reference values are given where possible. Although methodologies to automate CT pleural effusion segmentation^39^, ^40^, ^41^, ^42^ or decrease qualitative size estimation variability^43^ have been described, we found only one limited report of pleural effusion segmentation interobserver variability^39^ against which we can compare our results (despite extensive PubMed searches).

#### 2.C.1 Volumetric Dice similarity coefficient

The DSC measures interobserver agreement and ranges from 0 to 1, where 0 indicates no agreement and 1 indicates perfect agreement. In the context of image segmentation, the DSC relates the overlap between two segmentations to their total volumes; mathematically, this is twice the shared volume divided by the sum of their total volumes. No consensus dictates what constitutes a “good” DSC because the DSC is sensitive to the volume of the target structure. The American Association of Physicists in Medicine (AAPM) Task Group 132 notes that the contouring uncertainty of a structure can be expected to be a DSC of 0.8–0.9, while cautioning that “very large or very small structures may have different expected DSC values for contour uncertainty.”^44^ For lung segmentation, DSC interobserver variability between three medical physicist organizers of the 2017 AAPM Thoracic Auto‐Segmentation Challenge was reported as 0.956 ± 0.019 and 0.955 ± 0.019 for the left and right lungs, respectively.^45^ For pleural effusion segmentation, Yao et al.^39^ reported mean DSC observer variability between a research fellow, the same fellow months later, and an image processing technologist to be about 0.73. Comparisons against this reference value should be made cautiously because it was calculated for only 12 CT scans with unspecified pleural effusion volumes.

#### 2.C.2 Cohen’s Kappa coefficient

The Cohen’s kappa (κ) coefficient measures interobserver reliability for qualitative observations with mutually exclusive classifications. We computed κ between expert reviewer segmentations by treating each voxel as a qualitative datum. We transformed the segmentations to numerical arrays where each array value assumed one of two binary values depending on whether the reviewer included the voxel as part of the target structure. κ is similar to the DSC in its computation of interobserver agreement, but also assesses a likelihood of chance agreement and penalizes accordingly. Like the DSC, κ generally ranges between 0 and 1, although a result < 0 is possible. Results >0.6 are generally considered good and greater than 0.8 are considered very good,^46^ although like the DSC, κ inflates and deflates artificially depending on the target’s volume.

#### 2.C.3 Surface Dice similarity coefficient

Recognizing the limitations of the traditional DSC with respect to volume, Nikolov et al.^32^ introduced a novel way to compute Dice's coefficient called the surface DSC. As its name suggests, the sDSC's inputs are segmentation surfaces rather than volumes. A primary advantage of the sDSC over the traditional DSC is increased robustness to segmentation size variation. The sDSC is not agnostic to size, but it inflates less dramatically with size than the volumetric DSC. The sDSC computation accepts a tolerance parameter whereby differences between two surfaces can be tolerated as the same surface. We set this parameter equal to zero in order to capture all differences between physician segmentations. Because the sDSC is novel, reference values for this metric are not yet widely reported. However, Vaassen et al.^47^ calculated the sDSC between automatically generated and radiotherapist‐corrected lung segmentations and reported the majority of values to be greater than 0.85. Reasoning that inter‐physician agreement ought to be at least as good as autosegmentation‐radiotherapist agreement, we suggest 0.85 as an acceptable mean reference value for thoracic cavity segmentations.

#### 2.C.4 Hausdorff distance

In contrast to the DSC, κ, and sDSC, which measure fractional overlap between segmentations, HDs are geometric distances between segmentation surfaces. Larger HDs signify worse interobserver agreement. Computing a HD requires determining the minimum distances from every point on the surface of segmentation A to every point on the surface of segmentation B, and the same from B to A, and arranging them in ascending order. We report the 95HD, which is the value at the 95th percentile of the ordered minimum distances. 95HD interobserver variability reported by the AAPM Thoracic Auto‐Segmentation Challenge was 5.17 ± 2.73 and 6.71 ± 3.91 for left and right lung segmentations, respectively.^45^

#### 2.C.5 Average surface distance

The directed ASD from segmentation A to segmentation B is the average of the minimum distances from every point in the surface of A to every point in the surface of B. The directed ASD from B to A is calculated similarly. We report a symmetric ASD value that averages the two directed ASDs. Symmetric ASD interobserver variability reported by the AAPM Thoracic Auto‐Segmentation Challenge was 1.51 ± 0.67 and 1.87 ± 0.87 for left and right lung segmentations, respectively.^45^

#### 2.C.6 Expert reviewers

**Fig. 3:**
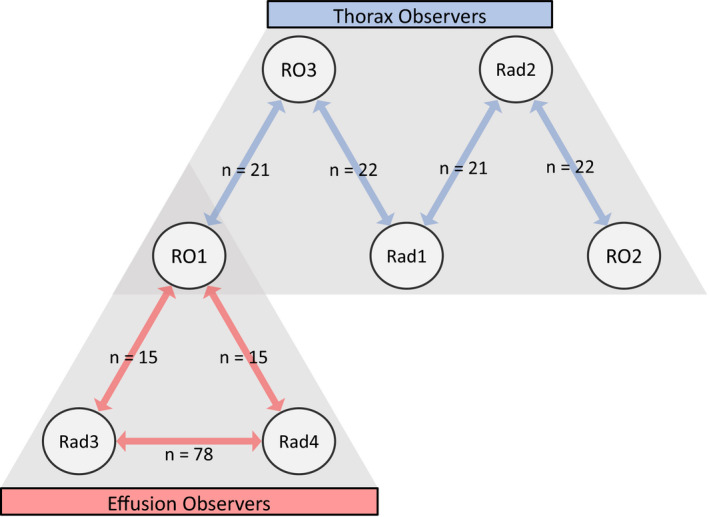
A schematic of interobserver comparisons, with the number of segmentation cases shared between observer pairs given as n. All 78 pleural effusion segmentations were reviewed and as necessary corrected by two radiologists: Rad3 and Rad4. A subset of 15 pleural effusion segmentations were also reviewed and corrected by RO1. In contrast, not all 402 thoracic cavity segmentations were reviewed by two physicians. Rather, four subsets of 21 or 22 thoracic cavity segmentations were randomly selected for dual review. All members of a given subset were exclusive to that subset. [Color figure can be viewed at wileyonlinelibrary.com]

Expert reviewers consisted of four radiologists and three radiation oncologists with varying years of experience (Table I). All thoracic cavity segmentations were reviewed by at least one expert, and randomly selected, unique subsets were reviewed by two. All pleural effusion segmentations were reviewed by at least two experts and a subset was reviewed by three. Relationships between reviewers are illustrated in Fig. 3. Median and minimum values for each spatial similarity metric for each reviewer pair are given in Table II. Pairwise Mann‐Whitney *U* tests^48^ conducted between reviewer pair distributions suggest that they are significantly different from one another for all metrics (*P* < 0.001). The following paragraphs discuss how interobserver variability in our study compares with variability described in other studies.

**Table I:** Seven radiologists (Rad) and radiation oncologists (RO) collaborated to review and correct 402 thoracic cavity segmentations and 78 pleural effusion segmentations delineated by a fourth‐year medical student.

Expert reviewer	Years of experience
Rad1	4
Rad2	2
Rad3	1
Rad4	3
RO1	4
RO2	11
RO3	5

**Table II:** Median and minimum values for Dice similarity coefficient (DSC), surface DSC, κ, 95HD, and symmetric ASD spatial similarity metrics calculated between paired physician segmentations. The distributions for each observer pair are significantly different from one another for all metrics (paired Mann‐Whitney *U* test^48^ *P* < 0.001). However, interobserver variability between pairs of physician‐vetted segmentations is generally acceptable. Median DSC, 95HD and symmetric ASD values for thoracic cavity segmentations are comparable to mean interobserver variability values reported by the 2017 AAPM Thoracic Auto‐Segmentation Challenge for lung segmentations.^45^ In general, pleural effusion segmentation interobserver agreement is also acceptable but more variable, reflecting both variation in pleural effusion size and inclusion or exclusion of trace pleural fluid.

Metric		Pleural effusions			Thoracic cavities			
		RO1‐Rad3	RO1‐Rad4	Rad3‐Rad4	RO1‐RO3	Rad1‐RO3	Rad1‐Rad2	RO2‐Rad2
Conformality metrics (unitless)								
DSC	Med	0.81	0.85	0.93	0.99	1.00	1.00	1.00
Min	0.10^a^	0.26	0.20	0.96	0.91^a^	0.99	0.97	
sDSC	Med	0.62	0.77	0.87	0.94	0.98	0.98	1.00
Min	0.20^a^	0.32	0.21	0.73^a^	0.82	0.94	0.88	
Kappa	Med	0.81	0.85	0.93	0.99	0.99	0.99	0.99
Min	0.10^a^	0.26	0.20	0.96	0.90^a^	0.99	0.97	
Surface distance metrics (mm)								
95HD	Med	24.00	21.65	5.31	1.95	0.00	0.00	0.00
Max	127.83	127.80	161.48^a^	11.35	55.11^a^	0.98	24.82	
ASD	Med	1.82	2.45	0.79	0.25	0.05	0.03	0.00
Max	23.53	22.39	33.49^a^	1.01	5.78^a^	0.12	1.68	

^a^ Select cases with extreme spatial similarity metric values are explored visually in Fig. 6.

**Fig. 4:**
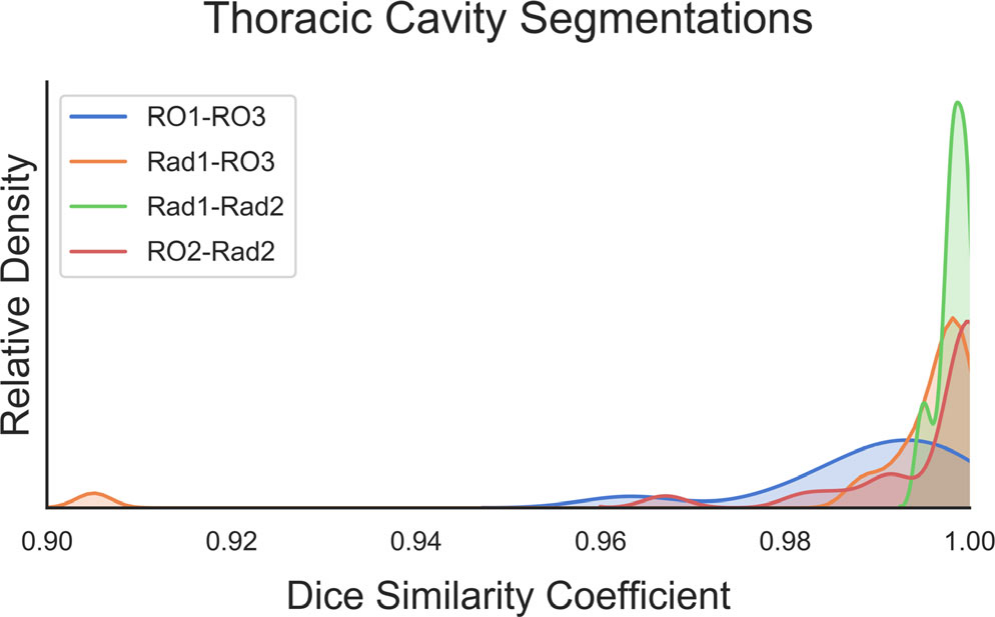
Dice similarity coefficient distributions reveal consistently strong agreement (>0.98) between paired, independently vetted radiologist and radiation oncologist thoracic cavity segmentations. Colored curves are kernel density estimates of DSC distributions. Note that Figs. 4 and 5 do not share the same x axis limits; the difference in DSC distributions in Figs. 4 and 5 is at least partially an artifact of the difference in average volume between thoraces and effusions. [Color figure can be viewed at wileyonlinelibrary.com]

In general, thoracic cavity segmentation pairs enjoyed a good level of agreement. Gauged by DSC, 95HD, and symmetric ASD spatial similarity metrics, thoracic cavity segmentation interobserver variability was similar to reported interobserver variability for lung segmentation^45^ and similar to values achieved by state‐of‐the‐art deep learning architectures trained for lung autosegmentation.^19^ Spatial similarity metrics improved with each iteration of corrections. For example, DSC values calculated between initial automated segmentations and substantially corrected medical student segmentations were relatively low (DSC_min_ = 0.353, DSC_med_ = 0.958, IQR = 0.041). In contrast, the DSC was never <0.9 between any pair of physician‐corrected contours, and the median DSC for each interobserver distribution approximated 0.99 (Fig. 4).

**Fig. 5:**
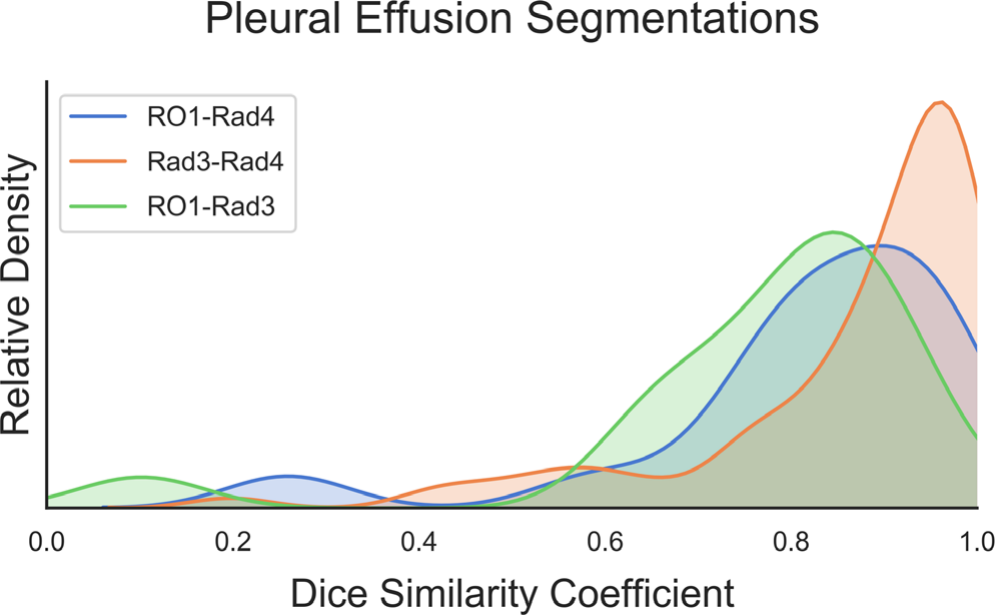
Dice similarity coefficient (DSC) distributions indicate good agreement (>0.8) between most paired, independently vetted radiologist and radiation oncologist pleural effusion segmentations. Interpretation of this result should respect that DSC values calculated on trace pleural effusions are sensitive to variation between segmentations on the order of only a few voxels. As in Fig. 4, colored curves are kernel density estimations of DSC distributions. [Color figure can be viewed at wileyonlinelibrary.com]

Pleural effusion segmentation interobserver agreement was also consistent, although the distributions of conformality metrics and surface distance metrics generally suggest lower agreement than for thoracic cavity segmentations. Medians DSCs (Fig. 5) compare favorably with Yao et al.’s^39^ mean DSC interobserver variability estimate of 0.73, but we again note that the sample in this study was small and the pleural effusion volumes unspecified. Knowing the pleural effusion volume distribution matters because wide variation in conformality spatial similarity metrics can be partly explained by the spread of pleural effusion volumes. Surface distance metrics can also be influenced by pleural effusion spatial spread (i.e., large distances separating effusion pockets). In a preprint analytic study that uses PleThora,^49^ we determined the median right and left pleural effusion volumes to be respectively 58.57 cm^3^ (IQR 30.31–113.7 cm^3^) and 50.85 cm^3^ (IQR 25.01–142.5 cm^3^). These are an order of magnitude smaller than the mean pleural effusion volumes described in some pleural effusion autosegmentation methodologic studies,^40^ which highlights the unique character of this dataset.

**Fig. 6:**
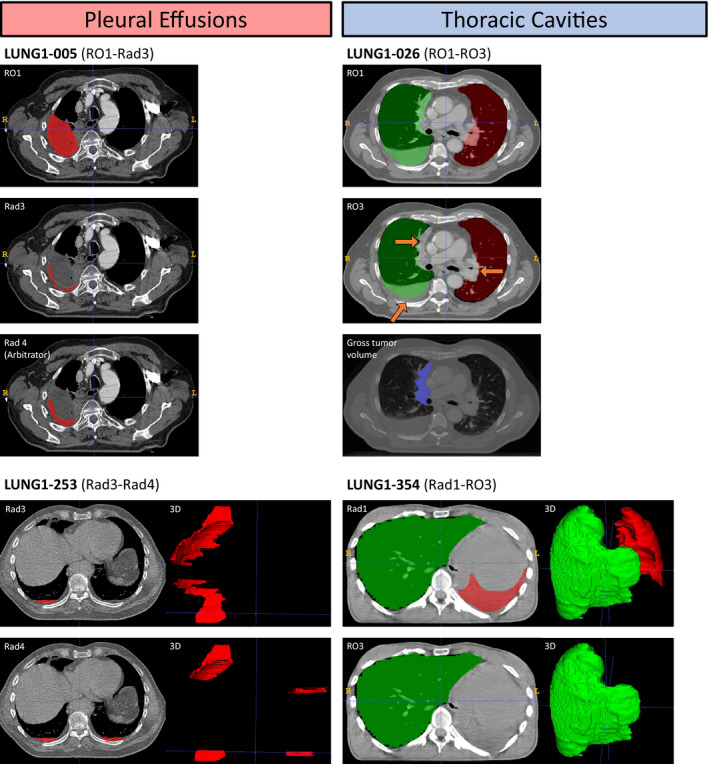
A visual exploration of physician‐corrected segmentation pairs with the least interobserver agreement. Case LUNG1‐005 accounts for the worst Dice similarity coefficient (DSC), surface DSC, and κ values between any pair of pleural effusion segmentations. RO1 mistook atelectatic lung for effusion, but Rad3 and Rad4 did not. Case LUNG1‐253 accounts for the worst 95HD and symmetric ASD values between any pair of pleural effusion segmentations. Rad3 and Rad4 varied in how much trace pleural fluid they chose to segment. This exposes a weakness in our pleural effusion segmentation methodology because we did not decide at projection initiation whether or to what extent trace pleural fluid should be part of the segmentation. Case LUNG1‐026 accounts for the worst sDSC value between any pair of thoracic cavity segmentations. RO3 failed to segment the full extent of peri‐mediastinal primary gross tumor volume, right effusion, and left hilum (orange arrows). Case LUNG1‐354 accounts for the worst DSC, κ, 95HD, and symmetric ASD values between any pair of thoracic cavity segmentations. RO3 erroneously excluded a collapsed left lung. [Color figure can be viewed at wileyonlinelibrary.com]

Notwithstanding that interobserver agreement was generally very good, a few segmentation cases demonstrated markedly poorer agreement than others (Fig. 6). RO1 and Rad3’s pleural effusion segmentations for case LUNG1‐005 yielded the worst DSC and κ values of any interobserver comparison. Visual inspection reveals a large posterior density that RO1 classified as pleural effusion but Rad3 excluded as an atelectatic lung segment. In this case, Rad4’s segmentation arbitrates that the density is not effusion. The segmentation we made publicly available through TCIA in our first version of the dataset was only Rad4’s segmentation, but all reviewer segmentations were made available in the second version. Rad3 and Rad4’s pleural effusion segmentations for case LUNG1‐253 suffered the highest 95HD value of any interobserver comparison. Here, 2D and 3D visual inspection reveals substantial disagreement respecting the superior‐inferior and lateral extents of a thin layer of pleural fluid. We did not prospectively define guidelines for whether trace pleural fluid should or should not be segmented, which in hindsight we acknowledge is a limitation of our pleural effusion segmentation methodology. By contrast, the worst discrepancies between thoracic cavity segmentations are easily resolved by our segmentation guidelines. Thoracic cavity segmentations for LUNG1‐026 suffered the worst sDSC interobserver variability, which can be attributed to the secondary physician reviewer’s erroneous neglect of the full extent of tumor, left hilum, and right pleural effusion. Similarly, the secondary reviewer erroneously excluded a left collapsed lung for case LUNG1‐354, which was the thoracic cavity segmentation case with the worst DSC, κ, 95HD, and symmetric ASD. The thoracic cavity segmentations we made publicly available for these cases in the first version of the dataset were the primary reviewer’s segmentations, but all reviewers’ segmentations were made available in the second version (after correcting the aforementioned errors).

### 2.D NSCLC‐radiomics collection update

The NSCLC‐Radiomics collection was updated on 10/23/2019, featuring new “RTSTRUCT” segmentations for all 422 cases, including revised GTVs in some cases. Our thoracic cavity segmentations were reviewed by radiologists or radiation oncologists who had access to “RTSTRUCT” files from the latest collection update. However, these segmentations were first delineated by a medical student at a time when only earlier versions of the “RTSTRUCT” files were available. In contrast, pleural effusion segmentations were all delineated with input from the latest “RTSTRUCT” files.

## 3 DATA FORMAT AND USAGE NOTES

### 3.A Data and metadata repository

In keeping with findable, accessible, interoperable, re‐usable (FAIR) data usage principles,^50^ all PleThora thoracic cavity and pleural effusion segmentations have been made available on TCIA at https://doi.org/10.7937/tcia.2020.6c7y‐gq39.^51^ Thoracic cavity segmentations are in a compressed NIfTI format, are named for their respective case and reviewer (e.g., “LUNG1‐001_thor_cav_primary_reviewer.nii.gz”), and are indexed in folders labeled after their respective NSCLC‐Radiomics collection cases (e.g., “LUNG1‐001”). Pleural effusion segmentations are likewise saved in a compressed NIfTI format and named for their respective case and reviewer (e.g., “LUNG1‐001_effusion_first_reviewer.nii.gz”). Many thoracic cavity and all pleural effusion segmentations were reviewed by two or more experts. In this dataset’s original TCIA publication, only primary reviewer segmentations were made available. However, all reviewers’ segmentations — primary, secondary, and tertiary — were made available in a recent dataset update (version 2). A spreadsheet entitled “Thorax and Pleural Effusion Segmentation Metadata” contains clinical and technical metadata pertaining to each segmentation or CT scan. It is hosted in the same repository as the segmentations. We strongly recommend users merge it with the NSCLC‐Radiomics spreadsheet “Radiomics Lung1.clinical‐version3‐Oct 2019.csv,” which provides ten columns of clinical data tied to each case. We provide Appendix 1 as a data dictionary to clarify the meaning of our spreadsheet’s column names.

### 3.B Baseline for deep learning model performance

To provide a performance baseline for researchers interested in using our thoracic cavity segmentations for deep learning model development, we trained and tested two U‐Net models, one based on 2D convolutional layers and one based on 3D convolutional layers. U‐Nets are common Convolutional Deep Neural Network architectures and form the basis of many deep learning algorithms for medical image segmentation.^52^, ^53^, ^54^, ^55^, ^56^

CT scans were preprocessed as follows: voxel intensities were clipped to a range of [−250, 0] Hounsfield units by reassigning voxels less than −250 to −250 and greater than 0 to 0, voxels were isotropically resampled to 1.7 mm in each dimension, and scans were cropped from the image center to 256 by 256 by 128 voxels. Segmentations were likewise resampled and cropped.

The models were trained using 316 of the 402 primary reviewer segmentations and tested with 86 secondary reviewer segmentations. The latter served as a test dataset because secondary and primary reviews were conducted independently. Nevertheless, because secondary and primary reviewers corrected the same template segmentation, we felt that corrected segmentations were likely to inherit similarities from the template that would bias the test dataset toward the training dataset. Therefore, we chose to exclude the 86 primary reviewer segmentations that corresponded to cases with a secondary reviewer from the training data. The model was initially trained end‐to‐end by fivefold cross validation. This permitted fine‐tuning of the hyperparameters (e.g., epochs, learning rate, batch size) without overfitting the external training set. The whole dataset split and secondary reviewer segmentations were recently made available through TCIA in an update (version 2) of the original dataset.

To build the 3D U‐Net model we used the architecture described by Çiçek et al.^53^ For each epoch the train/validation split was 80% train (252 scans) and 20% validation (64 scans). The batch size was 1 scan, the DSC was used to evaluate loss, and the learning rate was set initially to 0.001 and adapted by the Adam optimization algorithm.^57^ The DSC plateaued at 100 epochs. The mean DSC performance on the test dataset was 0.95 (standard deviation ± 0.05) and 0.95 (±0.04) for the left and right lungs, respectively. For reference, the DSC between the primary and secondary reviewer segmentations was 0.98 (± 0.11) and 0.98 (± 0.11) for the left and right lungs, respectively.

To build the 2D U‐Net model we used the same architecture as the 3D U‐Net but changed all 3D convolution and deconvolution operations to 2D operations. In this case, the algorithm was trained on 2D axial slices (i.e., 256 by 256 voxels) rather than whole volumes. Like the 3D model, the 2D U‐Net train/validation split was 80% train (32 256 slices) and 20% validation (8192 slices). The DSC plateaued at 60 epochs. The batch size was 64 slices, the DSC was used to evaluate loss, and the learning rate was set initially to 0.001 and adapted by the Adam optimization algorithm.^57^ The mean DSC performance on the test dataset was 0.94 (± 0.10) and 0.94 (± 0.10) for the left and right lungs, respectively.

## 4 DISCUSSION

To our knowledge, PleThora is the first public dataset of VOIs curated to capture all thoracic cavity pathologic change in patients with lung cancer, and the first public dataset of pleural effusion segmentations. The thoracic cavity segmentations are likely to be valuable to scientists and engineers who develop chest CT image processing pipelines that require robust methods to identify thoracic VOIs. We anticipate these segmentations to be a particularly useful addition to the corpus of training data for image processing pipelines, including the ones leveraging deep learning algorithms. Indeed, this project began as an effort to provide VOIs to study image feature symmetry between left and right thorax anatomy as a clinical outcomes predictor, building on previous work from our group that localized stroke cores by comparing and contrasting brain hemisphere information extracted by “symmetry‐sensitive convolutional neural networks.”^58^

Our pleural effusion segmentations are likely to be useful for investigating two questions surrounding a CT or PET/CT finding of pleural effusion: (a) the prognostic significance of pleural effusion in various cancer types,^59^ and (b) the capacity of CT to discriminate between benign and malignant effusions.^60^, ^61^, ^62^, ^63^ Regarding the first question, Ryu et al.^59^ showed that in small cell lung cancer with stage I–III disease, the presence of even minimal pleural effusion confers an increased risk of death. Investigating the prognostic relationship of pleural effusion presence in other cancer types would presumably be facilitated by deep learning pleural effusion segmentation algorithms, such as might be trained with datasets like ours. Regarding the second question, some CT findings (nodular, mediastinal, parietal, and circumferential pleural thickening) are classically considered to be reasonably specific but poorly sensitive discriminators of malignant effusion.^64^ Perhaps investigations into the value of pleural effusion quantitative imaging biomarkers for accomplishing this task could be fruitful. For example, Yang et al.^65^ reported that radiomics features extracted from lungs and pleura contained information capable of discriminating between patients with and without NSCLC dry pleural dissemination (AUC: 0.93; 95% CI: 0.891–0.968), which is a contraindication to primary tumor surgical resection that cannot always be detected by gross visualization. Our pleural effusion segmentations could enable other quantitative imaging biomarker studies. Importantly, our pleural effusion segmentations exclude primary tumor, as outlined in “RTSTRUCT” files, but users should not mistake this to mean that the effusions are necessarily benign. Microscopic tumor and macroscopic tumor below the threshold of radiologic detection are likely to exist in the effusions as delineated. It is also important to note that we failed to establish a guiding threshold for inclusion or exclusion of trace pleural fluid, an inconsistency that is reflected in several spatial similarity metrics we used to gauge interobserver variability. This stated, the effusion segmentations are still likely to be useful and may serve such research initiatives as correlating effusion parameters with clinical data available at the NSCLC‐Radiomics “Radiomics Lung1.clinical‐version3‐Oct 2019.csv” spreadsheet or investigating differences in radiomics features between effusion and primary tumor.

We acknowledge the limiting inconsistencies of human‐delineated segmentations, even those from trained radiologists or radiation oncologists. We also acknowledge intrinsic limitations in the metrics themselves. The DSC and κ are both artificially increased in large volumes, and the power of κ to penalize chance agreement is artificially decreased by the high number of true negatives in our segmentations (i.e., the high number of voxels that neither reviewer segmented as part of the target). We attempted to buffer this limitation by calculating and comparing sDSC values, which are less sensitive to variation in size. The 95 HD and symmetric ASD are not inflated by volume but are only snapshots of segmentations at their average (ASD) and near their worst (95HD) differences. Notwithstanding these limitations, we consider that the measures of interobserver variability obtained between radiologist and radiation oncologist reviewers justify acceptability of these segmentations for public use.

## 5 CONCLUSIONS

We describe PleThora, a dataset of 402 expert‐vetted thoracic cavity segmentations, 78 expert‐vetted pleural effusion segmentations, and corresponding clinical and technical metadata made available to the public through TCIA at https://doi.org/10.7937/tcia.2020.6c7y‐gq39.^51^ These segmentations have value for preprocessing steps in image analysis pipelines built for fundamental quantitative imaging tasks, including but not limited to pathologic lung segmentation, lesion detection, and radiomics feature extraction.

## CONFLICT OF INTEREST

The authors declare no conflict of interest relevant to this publication or the data therein described.

## Supporting information


**Appendix S1**. Data dictionary to the “Thorax and Pleural Effusion Segmentation Metadata” spreadsheet.
