# Supplementary material for: PleThora: Pleural effusion and thoracic cavity segmentations in diseased lungs for benchmarking chest CT processing pipelines
Source: Med Phys. 2020 Aug 28;47(11):5941–52. doi: 10.1002/mp.14424 (PMC7722027; doi:10.1002/mp.14424)
Supplement: Supplementary file 1 — Appendix S1. Data dictionary to the “Thorax and Pleural Effusion Segmentation Metadata” spreadsheet. [file MP-47-5941-s001.pdf]

Data Dictionary: descriptions of column names and their meanings as made available in the "Thorax and Pleural Effusion Segmentation Metadata" spreadsheet.

Column Names	Description
PatientID	"PatientID" is a case identifier inherited from NSCLC-Radiomics, and ties the data described in this manuscript to the data in that collection. Again, note that although 422 cases are present in the original collection, we present thoracic cavity segmentations for only 402 and pleural effusion segmentations for only 78.
Carcinoma Laterality	The laterality – right or left – of the primary tumor as determined by looking at the "GTV-1" structure in the NSCLC-Radiomics collection "RTSTRUCT" segmentation files.
GTV1	The gross tumor volume of the primary tumor in cm^3, determined from the "GTV-1" structure in the NSCLC-Radiomics collection "RTSTRUCT" segmentation files.
[GTV2 : GTV6]	Other gross tumor volumes as delineated in the "RTSTRUCT" segmentation files. Usually, these are nodal disease structures. Unlike the "GTV-1" structure, they are not consistently named nor always numerically named. Therefore, GTV2 to GTV6 should not be assumed to be the structure names. Rather, they were assigned in ascending order if the GTVs were numerically named (e.g. GTV2 → "gtv-2," GTV3 → "gtv-4," GTV4 → "gtv-7," etc.) and arbitrarily assigned if they were not (e.g. GTV2 → "gt_R," GTV3 → "gt_lt," etc.). No "RTSTRUCT" file had more than six GTV structures.
Tumor Location	Location of the primary tumor with respect to centrality. Centrality has not always been consistently defined by international associations nor clinical trial

	protocols[1]; we defined central tumors as within 2 cm of the proximal bronchial tree or mediastinum and extending up to 4 cm from these structures. Peripheral tumors were defined as beyond 2 cm from the proximal bronchial tree or mediastinum. Pan tumors were within the central zone but spanned beyond 4 cm from central structures into the peripheral zone. Central tumors were coded as 1, peripheral tumors as 2, and pan tumors as 3.
Effusion Event	The presence or absence of effusion in the CT scan. Effusion presence is coded as 1, and absence as 0.
Primary Effusion Reviewer	The radiologist who vetted and corrected the medical student's manual pleural effusion segmentations, and whose corrected segmentations comprise the dataset available through TCIA.
Secondary Effusion Reviewer	A second radiologist or radiation oncologist who independently corrected the medical student's manual pleural effusion segmentations.
Tertiary Effusion Reviewer	A third radiologist or radiation oncologist who independently vetted the medical student's manual pleural effusion segmentations.
Right Pleural Effusion Volume	The volume (in cm^3) of the right pleural effusion, if present.
Left Pleural Effusion Volume	The volume (in cm^3) of the left pleural effusion, if present.
RO1-Rad4 Effusion DSC	Dice similarity coefficient between independently corrected pleural effusion segmentations by a radiation oncologist and a radiologist. The calculation used to compute DSCs in this and the following two columns treats left and right effusions (if both are present) as a single label.

Rad3-Rad4 Effusion DSC	Dice similarity coefficient between independently corrected pleural effusion segmentations by two radiologists.
RO1-Rad3 Effusion DSC	Dice similarity coefficient between independently corrected pleural effusion segmentations by a radiation oncologist and a radiologist.
Thorax Recontour Time	Time (in minutes) required for the medical student to expand the UNet automated lung segmentation to include all the thoracic cavities bilaterally.
Auto-MS Thorax DSC	Dice similarity coefficient between the initial automated segmentation and a medical student's corrected segmentation, prior to further correction by a radiation oncologist or radiologist.
Primary Thorax Reviewer	The radiologist or radiation oncologist who vetted and corrected the medical student's thoracic cavity segmentations, and whose corrected segmentations comprise the dataset available through TCIA.
Secondary Thorax Reviewer	A second radiologist or radiation oncologist who independently corrected the medical student's thoracic cavity segmentations.
Right Thorax Volume	The volume (in cm^3) of the right thorax, exclusive of the mediastinum, but inclusive of primary tumor if this invaded the mediastinum or extrathoracic region.
Left Thorax Volume	The volume (in cm^3) of the left thorax, exclusive of the mediastinum, but inclusive of primary tumor if this invaded the mediastinum or extrathoracic region.
RO1-RO3 Thorax DSC	Dice similarity coefficient between independently corrected thoracic cavity segmentations by two radiation oncologists. The calculation used to compute

	the DSC in this and the following three columns treats left and right thoraces as a single label.
Rad1-RO3 Thorax DSC	Dice similarity coefficient between independently corrected thoracic cavity segmentations by a radiologist and a radiation oncologist.
Rad1-Rad2 Thorax DSC	Dice similarity coefficient between independently corrected thoracic cavity segmentations by two radiologists.
R02-Rad2 Thorax DSC	Dice similarity coefficient between independently corrected thoracic cavity segmentations by a radiation oncologist and a radiologist.
Dim X	Dimension of the CT scan and segmentation files along the x axis.
Dim Y	Dimension of the CT scan and segmentation files along the y axis.
Dim Z	Dimension of the CT scan and segmentation files along the z axis.
Voxel Space X	Voxel spacing of the CT scan and segmentation files along the x axis, in mm
Voxel Space Y	Voxel spacing of the CT scan and segmentation files along the y axis, in mm
Voxel Space Z	Voxel spacing of the CT scan and segmentation files along the z axis, in mm

Reference

1. Roesch J, Panje C, Sterzing F, et al. SBRT for centrally localized NSCLC - What is too central? *Radiat Oncol.* 2016;11(1):157. doi:10.1186/s13014-016-0732-5
